# Influence of heat shock and osmotic stresses on the growth and viability of *Saccharomyces cerevisiae* SUBSC01

**DOI:** 10.1186/s13104-015-1355-x

**Published:** 2015-08-23

**Authors:** Md. Sakil Munna, Sanjida Humayun, Rashed Noor

**Affiliations:** Department of Microbiology, Stamford University Bangladesh, 51 Siddeswari Road, Dhaka, 1217 Bangladesh

**Keywords:** Heat stress, Osmotic stress, *Saccharomyces cerevisiae*, Critical growth temperature, Budding yeasts, Viable and culturable cells

## Abstract

**Background:**

With a preceding scrutiny of bacterial cellular responses against heat shock and oxidative stresses, current research further investigated such impact on yeast cell. Present study attempted to observe the influence of high temperature (44–46 °C) on the growth and budding pattern of *Saccharomyces cerevisiae* SUBSC01. Effect of elevated sugar concentrations as another stress stimulant was also observed. Cell growth was measured through the estimation of the optical density at 600 nm (OD_600_) and by the enumeration of colony forming units on the agar plates up to 450 min.

**Results:**

Subsequent transformation in the yeast morphology and the cellular arrangement were noticed. A delayed and lengthy lag phase was observed when yeast strain was grown at 30, 37, and 40 °C, while at 32.5 °C, optimal growth pattern was noticed. Cells were found to lose culturability completely at 46 °C whereby cells without the cytoplasmic contents were also observed under the light microscope. Thus the critical growth temperature was recorded as 45 °C which was the highest temperature at which *S. cerevisiae* SUBSC01 could grow. However, a complete growth retardation was observed at 45 °C with the high concentrations of dextrose (0.36 g/l) and sucrose (0.18 g/l). Notably, yeast budding was found at 44 and 45 °C up to 270 min of incubation, which was further noticed to be suppressed at 46 °C.

**Conclusions:**

Present study revealed that the optimal and the critical growth temperatures of *S. cerevisiae* SUBSC01 were 32.5 and 45 °C, respectively; and also projected on the inhibitory concentrations of sugars on yeast growth at that temperature.

## Findings

Stress responses in bacterial cells and to some extent in the yeast cells have been well studied so far [1–18]. Abrupt changes in the environmental and physicochemical stimuli including temperature, pH, sugar/salt concentrations, the redox state, toxic compounds and nutrient exhaustion have been mostly found to elicit a battery of defending response by up-regulating the genes encoding heat shock proteins (HSPs) in bacterial cells [19–27]. Like bacteria, the heat shock response in *Saccharomyes cerevisiae*, the model experimental yeast species, has been also characterized by the rapid changes in their cellular physiology including the budding manner accompanied with the increased tolerance against elevated salt and sugar concentrations, and against reactive oxygen species (ROS) [1–4, 7, 9–11, 15, 18, 28–30]. In *S. cerevisiae*, heat-sensitivity is ordinarily prescriptive of defects in protein coding genes which are also essential for maintaining the cell viability [10, 19, 24, 31–34]. The coupling consequence of heat stress together with the osmotic shock has been found to influence the cellular degeneration along with the retardation in cell division in yeast cells [3, 7, 35].

Our earlier studies revealed the bacterial cellular adaptation in response to the heat shock and against the elevated amount of intracellular reactive oxygen species (ROS) [14, 17, 36–38]. However, the work on stress response in yeast cells is scarce in the local perspective. These led us to broaden the research interest in the yeast cells to assess the optimal and critical growth temperatures and further to investigate intensely the growth changes at the critical temperature accompanied with the simulated stressed condition of an ascending osmotic pressure. Thus, apart from our earlier experiments on bacterial stress responses, current study was designed to observe the stress response in *S. cerevisiae* SUBSC01 towards heat shock and elevated sugar concentrations. The key observation revealed that while at 45 °C the yeast strain could grow, conversely growth inhibition was noticed upon supplementation of high concentrations of sugars.

## Methods

On the basis of strain availability, laboratory stock cultures of *S. cerevisiae* SUBSC01 were used. Sabouraud Dextrose Agar (SDA) (Hi-Media Laboratories Pvt. Ltd., India), Sabouraud dextrose broth (SDB) (Difco Laboratories, Inc. USA) and Sucrose broth (SB) (Sigma-Aldrich Corporation, USA) were used. Pre-cultures were prepared by inoculating 5 ml SDB by a loopful of colony from the freshly prepared yeast culture plates, followed by incubation at 30, 32.5, 37 and 40 °C in static condition up to 72 h. The optical density at 600 nm (OD_600_) and the capability to form the colony forming units (CFUs) were monitored at the specific time intervals [13]. To determine the critical growth temperature, growth was monitored at 44, 45 and at 46 °C. For morphological observations, an aliquot of 5 µl from each of the culture suspension was removed at 90 min intervals [17, 39, 40]. For spot dilution tests, 1 ml of the culture suspension at same intervals was removed and serially diluted in 9 ml dextrose broth up to 10^−4^ [17]. An aliquot of 5 µl from each dilution was then spotted onto SDA plates following incubation at 32.5 °C for 24 h. To observe the osmotic effect on cell growth, different concentrations of dextrose including 0.04 g/l (1X), 0.12 g/l (3X), 0.2 g/l (5X), 0.28 g/l (7X), 0.36 g/l (9X) and sucrose, i.e., 0.02 g/l (1X), 0.06 g/l (3X), 0.1 g/l (5X), 0.14 g/l (7X), and 0.18 g/l (9X) were used. All experiments were conducted in triplicates. Statistical analysis regarding yeast growth was performed by determining the P value (~0.3) through t test. Standard deviations were also measured with the aid of statistical hypothesis testing [17].

## Results interpretation and analysis

### Optimal growth temperature of *Saccharomyces cerevisiae* (SUBSC01)

**Fig. 1:**
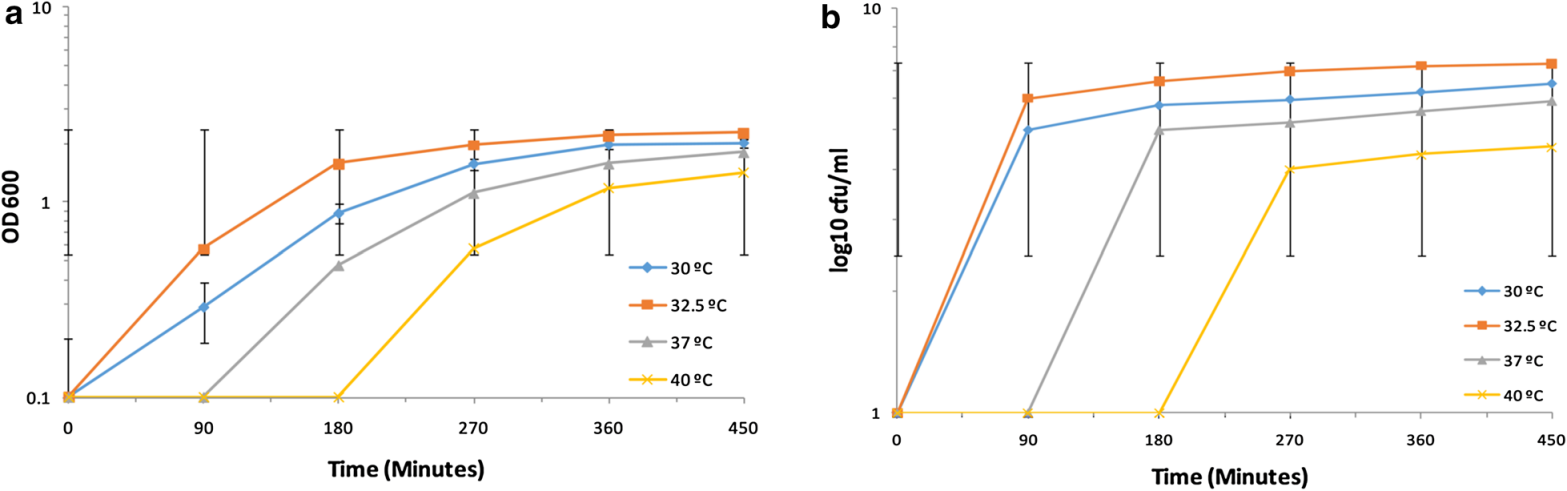
Assessment of the optimum temperature through the examination of growth of *Saccharomyces cerevisiae* SUBSC01in terms of **a** Optical Density at 600 nm (OD_600_) and **b** the formation of the colony forming units (CFUs). Cells were grown as stated in “Methods”, and aliquots were removed at the specific time intervals (90, 180, 270, 360 and 450 min) for the assay. A prolonged lag phase (~270 min) was observed, when cells were grown at 30, 37, 40 °C. Consequently, the optimum growth temperature of the laboratory stain *S. cerevisiae* SUBSC01 was estimated to be 32.5 °C

**Fig. 2:**
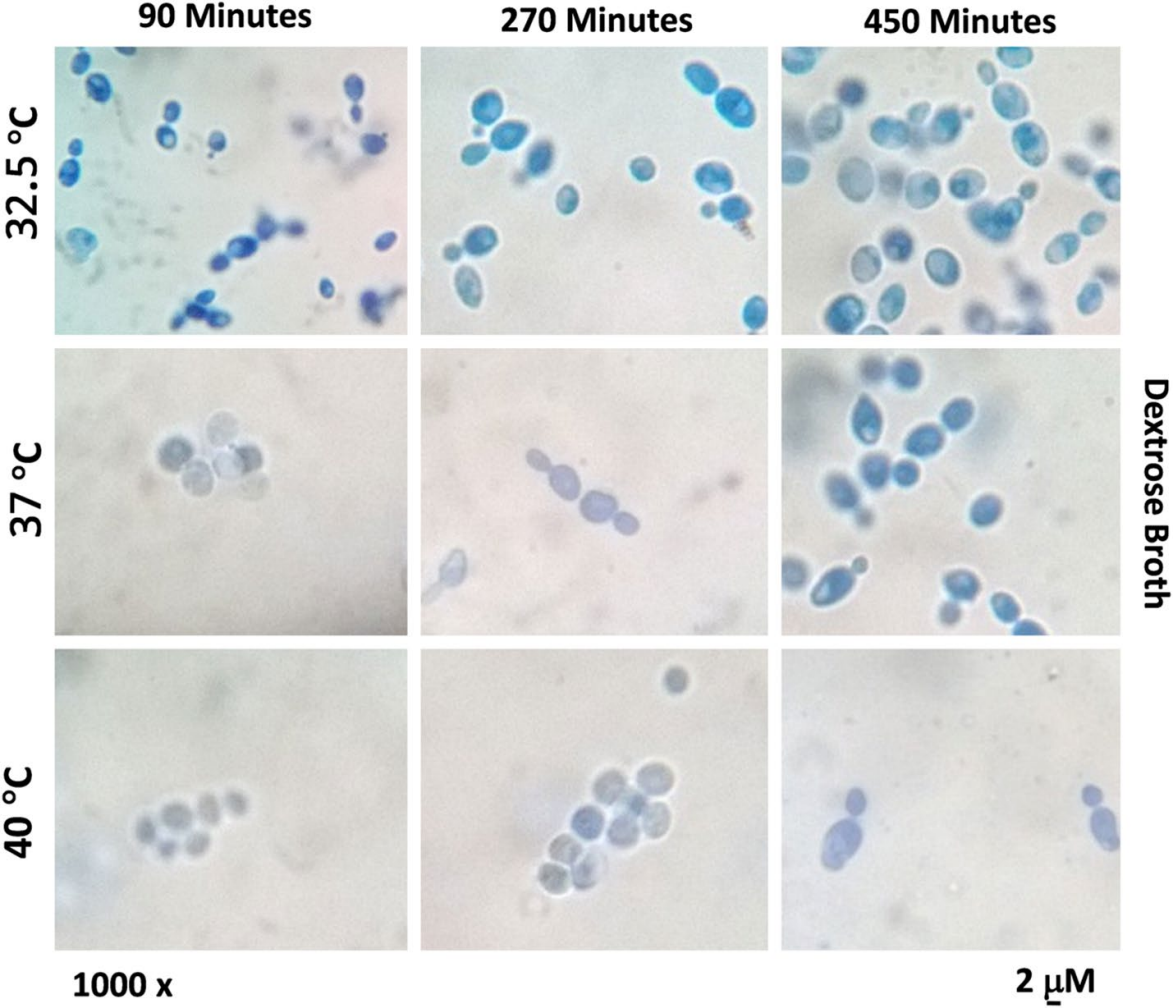
Morphological study of *S. cerevisiae* SUBSC01 cells at optimal (32.5 °C) and high (40 °C) temperatures. Cells were grown as stated in “Methods”, and aliquots were removed at 90, 270 and 450 min for the assay. Active budding yeast was observed under light microscope at 32.5 °C up to 450 min of incubation. Besides, stressed cells (cells without the cytoplasmic contents) were observed at 40 °C after 450 min of incubation

The optimal growth temperature for *S. cerevisiae* SUBSC01 was assessed through the measurement of OD_600_ and by counting the CFUs up to 450 min. After 90 min of incubation at 32.5 °C the cell number was found to increase rapidly by approximately 4 logs (Fig. 1), whereas at 30 °C such tendency was a bit slower (Fig. 1). Interestingly, compared with the growth state at 32.5 and 30 °C, a relatively lengthy lag phase (~270 min) was observed when cells were grown at 37 and 40 °C, possibly due to the requirement of longer time to cope with temperatures higher than the optimal growth condition (Fig. 1). Besides, under the light microscope, budding yeasts were observed after 90 min at 30 and 32.5 °C, whereas after 180 min such budding was observed at 37 °C. At 40 °C, the budding events were noticed after 270 min (Fig. 2). The optimal growth temperature for *S. cerevisiae* SUBSC01 was thus noted to be 32.5 °C. However, it is to be mentioned that earlier *S. cerevisiae* was found to exhibit optimal growth temperature between 25 and 35 °C as reported by the other groups [41–44].

### Critical growth temperature of *Saccharomyces cerevisiae* (SUBSC01)

**Fig. 3:**
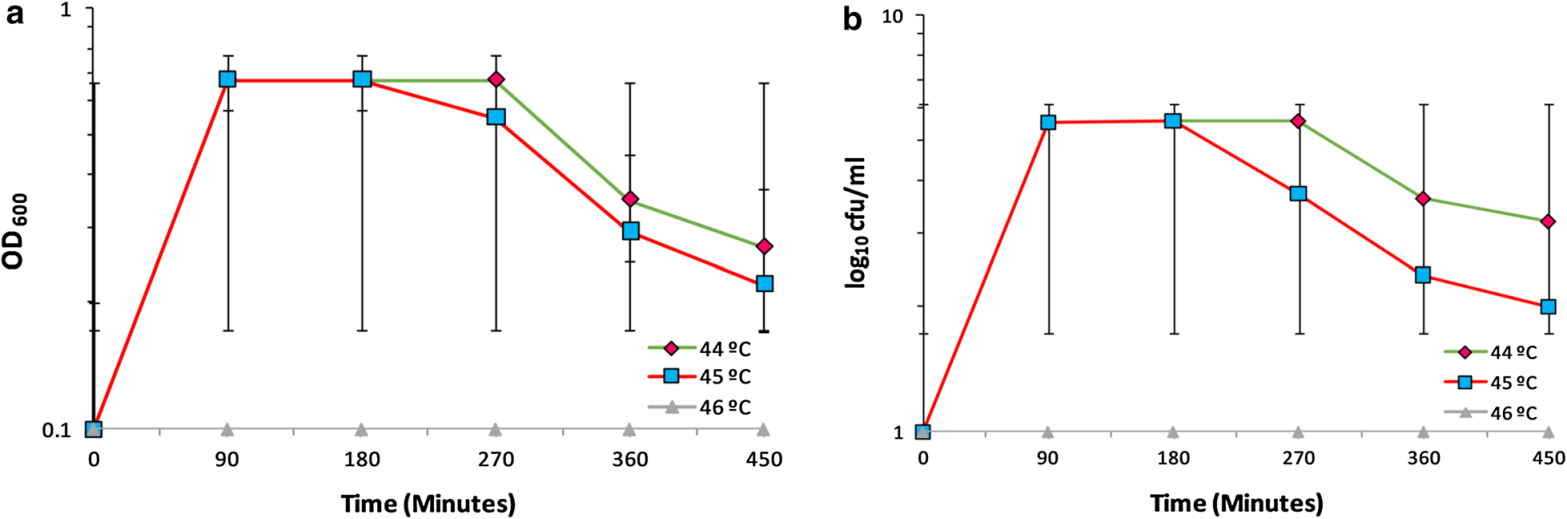
Effect of high temperatures (44–46 °C) on growth of *S. cerevisiae* SUBSC01: **a** impact on cell turbidity and **b** influence on the formation of CFUs. Cells were grown as stated in “Methods”, and aliquots were removed at the specific time intervals as indicated. A complete decline of both OD_600_ and CFUs was noticed at 46 °C. The critical temperature of *S. cerevisiae* SUBSC01 was recorded at 45 °C

**Fig. 4:**
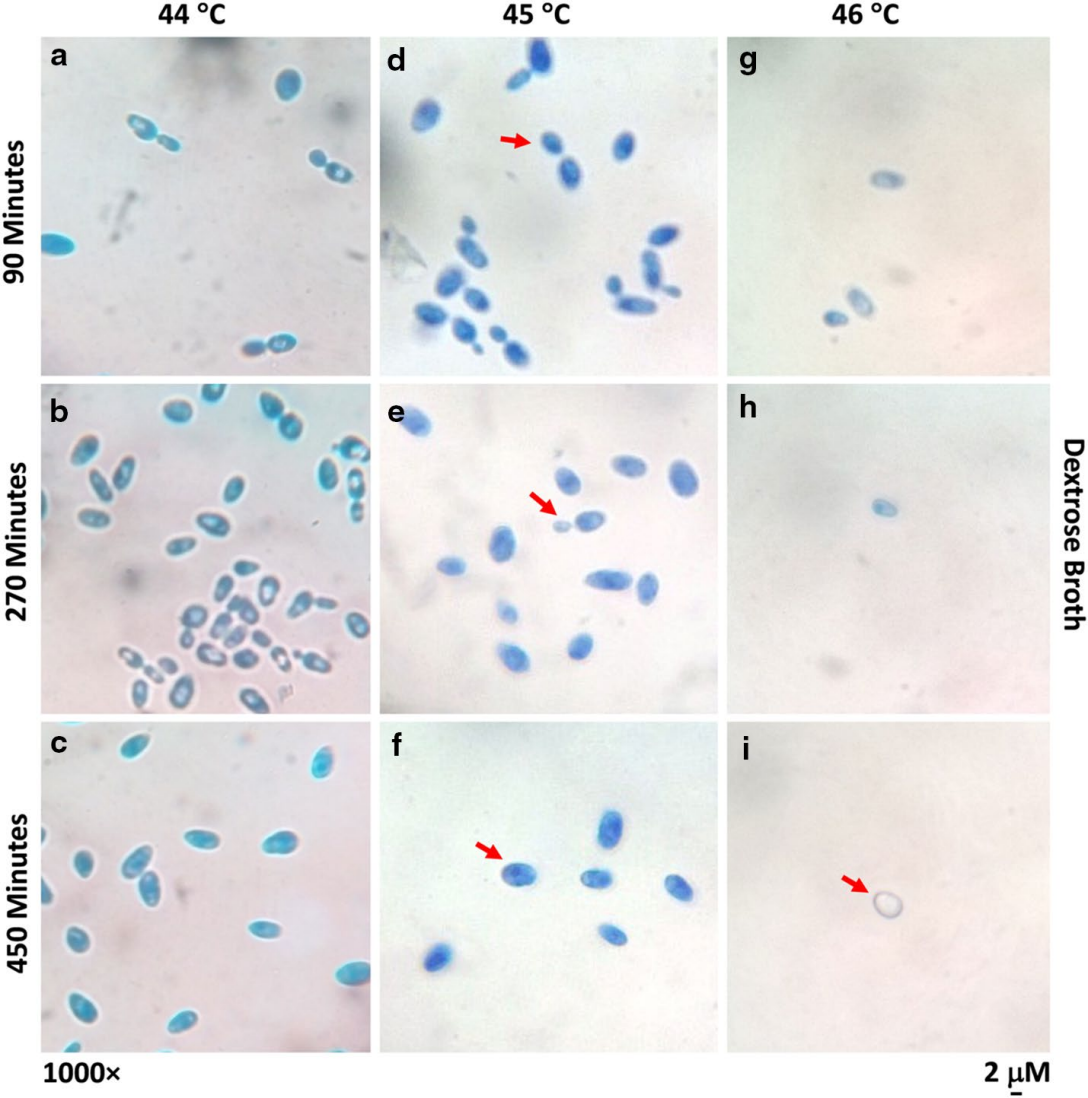
Observation of the morphological changes of S. cerevisiae SUBSC01 cells at 44 °C (**a**–**c**), 45 °C (**d**–**f**) and 46 °C (**g**–**i**). Cells were grown as stated in “Methods”, and aliquots were removed at the specific time intervals (90, 270 and 450 min). Interestingly, budding cells were found to be dormant at 45 °C (**d**–**f**). Also the cells were found to lose their cytoplasmic content when grown at 46 °C (**i**)

The ability of the yeast strain to grow at 40 °C led our interest further to examine the maximal growth temperature limit. While a sharp drop was observed in CFU and a relatively lower reduction in the cell turbidity was noticed at 44 and 45 °C after 180 and 450 min, respectively; notably an inclusive retardation of growth was also observed when cells were grown at 46 °C (Fig. 3). Interestingly, the budding yeasts were found to become dormant when cells were grown at 45 °C (Fig. 4d–f). In addition, all cells were found without the cytoplasmic contents at 46 °C (Fig. 4i).

An important physiological point is to ponder that unlike *Escherichia coli* cells, the yeast cells grown at high temperature did not exhibit the characteristics of cell lysis, possibly due to the comparatively rigid cell membrane and cell wall [13, 16]. However, as has been seen in the current study, the generation of the cells without the cytoplasmic contents due to the deletion of *rpoE* gene (encoding the RNA polymerase σ^E^) in the bacterium *Escherichia coli* W3110 has also been observed earlier through electron microscopy [16]. In the current study, such an observation of the yeasts cells lacking the cytoplasmic contents under the stressed condition has further drawn the interest on the global impact of heat shock on microorganisms at the cellular level, and led us further to cross-check the expected loss of the cell viability at high temperature by means of the spot dilution tests [17, 38]. In consistent with the results from the growth assessment, all yeast cells were found to lose the culturability completely at 46 °C as observed through the spot dilution tests (results not shown). Hence the critical growth temperature of this strain was recorded at 45 °C.

### Growth retardation of *S. cerevisiae* SUBSC01 at critical temperature accompanied with an ascending osmotic shock

**Fig. 5:**
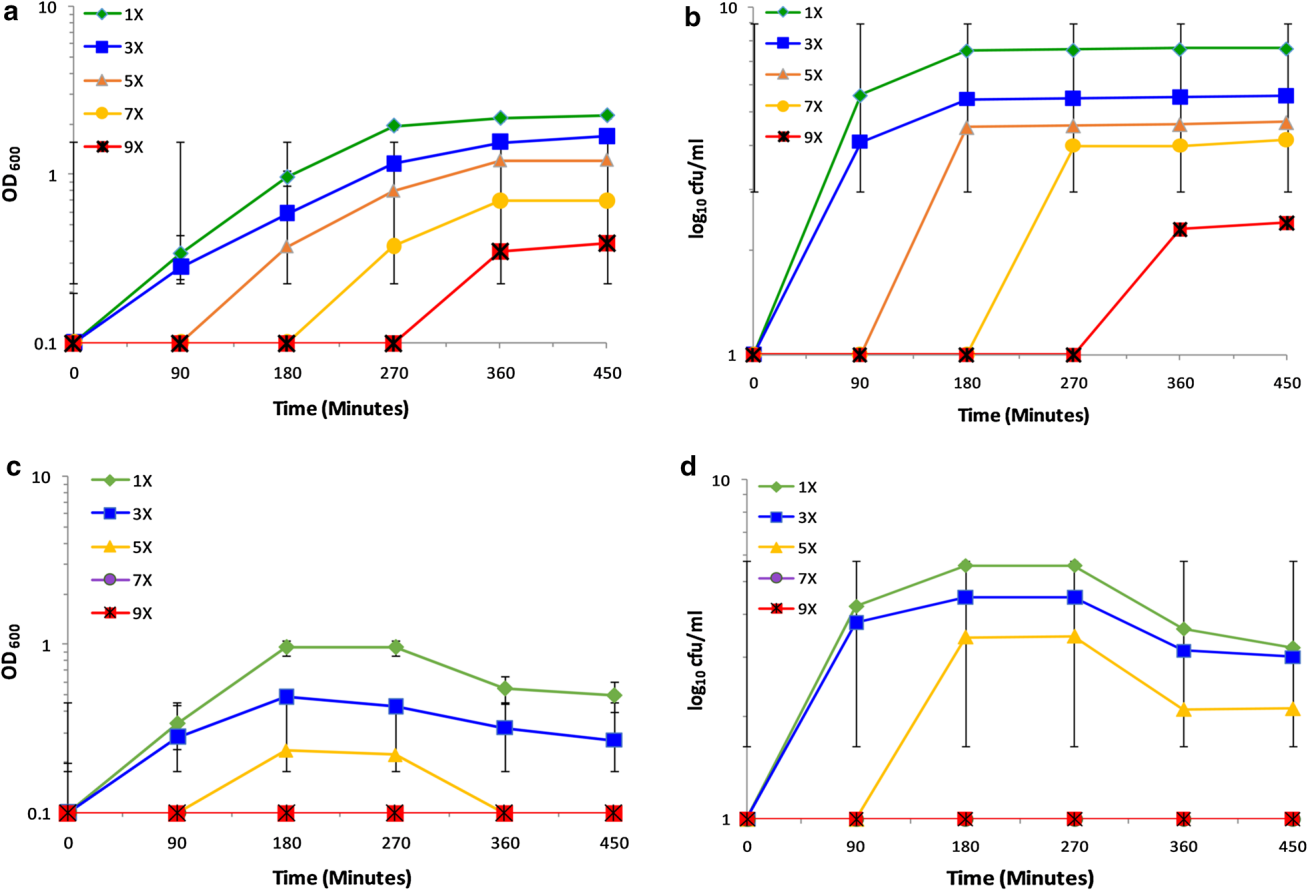
Growth retardation of *S. cerevisiae* SUBSC01 at 32.5 and 45 °C with different dextrose concentrations. Cell culturability was assessed through OD_600_ and the formation of CFUs at 32.5 °C (**a**, **b**), and at 45 °C (**c**, **d**) with different dextrose concentrations, i.e., 0.04 g/l (1X), 0.12 g/l (3X), 0.2 g/l (5X), 0.28 g/l (7X), 0.36 g/l (9X). Cells were grown as stated in “Methods”, and aliquots were removed at the specific time intervals of 90, 180, 270, 360 and 450 min. A complete elimination of culturable cells were observed at 45 °C with 7X and 9X dextrose concentrations (**c**, **d**)

**Fig. 6:**
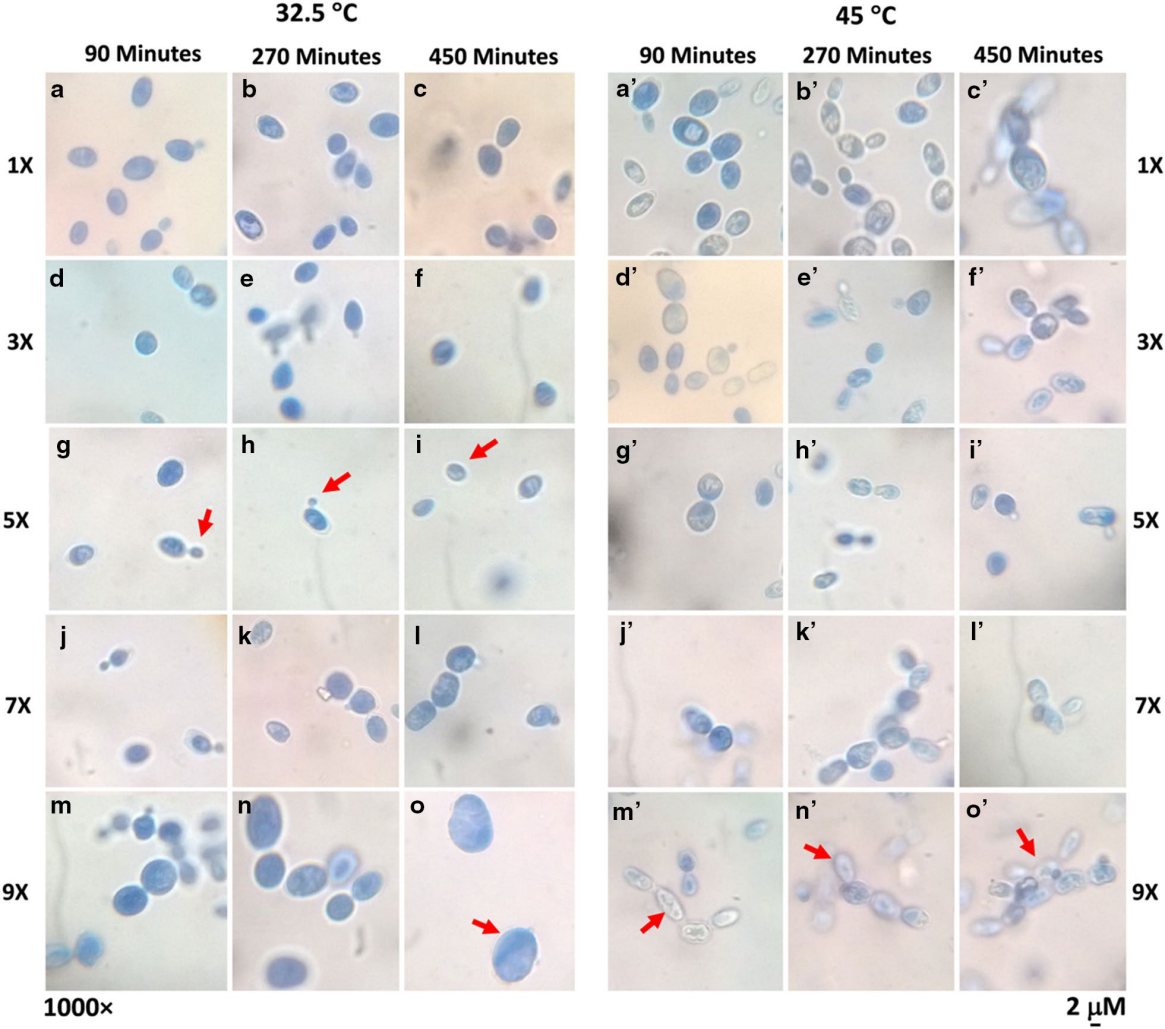
Morphological study of *S. cerevisiae* SUBSC01 at 32.5 °C (**a**–**o**) and 45 °C (**a’**–**o’**) upon osmotic stresses. Cells were grown as stated in “Methods”, aliquots were removed at 90, 270 and 450 min. Budding cells became dormant at 32.5 °C with 5X (0.14 g/l) dextrose concentration (**g**–**i**), and were found to be thickened at 9X dextrose concentration (**o**). All cells lost cytoplasmic contents at 45 °C with 9X (0.18 g/l) dextrose concentrations (**m**–**o**)

In order to achieve the complete stress response consequences of *S. cerevisiae* SUBSC01 upon critical temperature, different levels of osmotic pressure were simulated onward. A relatively lengthy lag phase (~360 min) was observed in both OD_600_ and CFU in compliance with the extended dextrose concentrations at 32.5 °C (Fig. 5a, d). Approximately 4 log CFU/ml was found to be abolished in 7X and 9X dextrose concentrations. Besides, even capable of growing at 45 °C, cells were found to lose their culturability completely at this temperature when the culture medium was supplemented with extremely high (9X) dextrose concentration (Fig. 5c, d). Earlier investigation also showed that yeast cells may exhibit an immediate growth arrest when exposed to an increase in external osmolarity [45]. The prolonged lag phase (~360 min) in the culturable cells (Fig. 5) led us to examine the probable morphological changes and impairments in the stressed yeast cells afterward. Interestingly the budding cells were found to become quiescent at the optimal temperature with 5X dextrose concentration (Fig. 6g–i), and additionally cells were also found to be thickened (Fig. 6o) at 9X dextrose concentration (Fig. 6m–o). In cohesion to the current findings, previously, the cell volume of *S. cerevisiae* was also found to expand at 48 h of incubation periods upon osmotic stress [46]. However, a huge number of cells loosing the cytoplasmic contents were observed at 45 °C with extreme high (9X) dextrose concentration (Fig. 6m′–o′). Consistently, in the absence of osmotic imbalances no stressed cells (cells without the cytoplasmic contents) were observed at 45 °C.

As stated earlier, *S. cerevisiae* SUBSC01 exhibits approximately 360 min long lag phase at 32 °C and complete growth suppression at 45 °C due to osmotic shock. Elimination of such growth was further supported by the appearance of the stressed cells as seen under the microscope (Fig. 6m′–o′). Such results led us to further cross check the stressed physiology of the cells through spot test. At 32.5 °C in different dextrose concentrations (1X–9X), cells were found culturable through spot dilution tests (results not shown). At 45 °C under high (9X) dextrose concentration, cells were found to lose their culturability completely. Earlier research found that the activity of β-fructofuranosidase (*SUC2*) of *S. cerevisiae*, which is liable for sucrose degradation; might be repressed by the increased osmotic pressure [28, 32]. This is to be mentioned that when the cells were grown at 32.5 °C in different sucrose concentrations, all were found to grow after a certain incubation period (results not shown). Nevertheless, the current investigation clearly unraveled the heat stress responsive events in *S. cerevisiae* SUBSC01, which is comprehensible with the existing knowledge on yeast growth phases and stress physiology.

The revelation of the temperature tolerance of yeast cells as revealed from the current study is consistent with the recent findings [15, 21, 47]. Indeed, deviation in temperature is a general stress encountered by yeast cells [47]. *S.* *cerevisiae* is well known to generate the protective transcriptional programs in response to elevated temperatures [15, 21]. However, those studies mostly showed the temperature tolerance at around 37 °C while the current study clearly showed that the yeast strain studied here could withstand up to 45 °C. Besides, the findings of the critical growth temperature besides the optimal condition, sugar tolerance level, and a bit interestingly the observation of prolonged lag phase at high temperatures may be of significance in the field of yeast physiology. Presented results may provide further general information on the triggering phase of heat shock events in yeast cells. Further studies regarding the expressional analyses of the stress responsive genes would unveil the involvement of the necessary regulons and chaperons required for the stress defense mechanism in the *S. cerevisiae* SUBSC01.
